# Breastfeeding and mental health in adulthood: A birth cohort study in Brazil

**DOI:** 10.1016/j.jad.2016.05.055

**Published:** 2016-09-15

**Authors:** Christian Loret de Mola, Bernardo Lessa Horta, Helen Gonçalves, Luciana de Avila Quevedo, Ricardo Pinheiro, Denise Petrucci Gigante, Janaína Vieira dos Santos Motta, Fernando C. Barros

**Affiliations:** a Postgraduate Program in Epidemiology, Federal University of Pelotas, Brazil; b Nursing Department, Federal University of Pelotas, Brazil; c Health and Behavior Postgraduate Program, Universidade Católica de Pelotas – UCPEL, Pelotas, RS, Brazil

**Keywords:** Depression, Mental health, Anxiety, Breastfeeding, Cohort, Brazil

## Abstract

**Background:**

Breastfeeding is negatively associate with behavioral and internalization problems, psychological stress, and depressive/anxiety symptoms. However, studies evaluating specific mental health disorders are scarce. We aimed to assess the association between breastfeeding and mental health outcomes in young adults.

**Methods:**

In 1982, hospital deliveries in Pelotas (Southern Brazil) were identified; liveborns were examined and their mothers interviewed (n=5914). Information on breastfeeding was collected in early childhood. In 2012–13, at 30 years of age, we used the Mini International Neuropsychiatric Interview (MINI) for the diagnosis of major depression (MD), generalized anxiety disorder (GAD) and social anxiety disorder (SAD). In addition, we used the Beck Depression Inventory (BDI-II) and the Self-reported Questionnaire (SRQ-20), to evaluate depressive symptoms severity and common mental disorders (CMD), respectively. We used multivariable regression models to evaluate the association between breastfeeding and mental health outcomes.

**Results:**

We evaluated 3657 individuals. Prevalence of CMD, MD, GAD and SAD was 24.3%, 7.9%, 12.7% and 3.6%, respectively. In multivariable models the odds of having a more severe case of depression (BDI-II) was smaller among those breastfed for 6 or more months (OR=0.69 95%CI [0.53–0.89]). We observed a similar pattern for MD and CMD, however, confidence intervals included the reference.

**Limitations:**

We had no information on home environment characteristics during childhood. Lack of power and a small effect size could explain why we did not detect an association between breastfeeding and MD.

**Conclusion:**

Breastfeeding reduced the odds of having more severe depressive symptoms.

## Highlights

• Breastfeeding reduced the odds having more severe depressive symptoms in young adults.
• The pattern of association was similar in all depressive like outcomes.
• The effect of breastfeeding over major depression might be small.
• Socioeconomic residual confounding is unlikely in this cohort.

## 1 Introduction

Besides the short-term benefits of breastfeeding, evidence shows that been breastfed for a longer time has long-term consequences. It has been reported that breastfeeding may reduce the likelihood of obesity and type-2 diabetes. (Horta et al., 2015a, Kelishadi and Farajian, 2014), increase performance in cognitive tests (Horta et al., 2015a) and it is associated with a higher income in adulthood (Victora et al., 2015).

In addition, breastfeeding is negatively associated with behavioral and internalization problems (Hayatbakhsh et al., 2012, Heikkila et al., 2011, Liu et al., 2006, Liu et al., 2014, Yi et al., 2005) as well as psychological stress (Montgomery et al., 2006) and other mental health outcomes like depressive and anxiety symptoms (Hayatbakhsh et al., 2012, Oddy et al., 2010, Reynolds et al., 2014), major depression (Allen et al., 1998, Peus et al., 2012a, Peus et al. 2012a) and attention deficit disorder (ADHD) (Mimouni-Bloch et al., 2013, Stadler et al., 2015).

Merjonen et al. (2010) reported that breastfeeding might decrease the risk of depression possibly associated with the C/C genotype of the estrogen receptor 1 gene. However, other studies have failed to observe an association between breastfeeding and later mental health (Anselmi et al., 2008, Kramer et al., 2008, Kwok et al., 2013, Lind et al., 2014).

Most of the studies on the association between infant feeding and mental health have been done in high-income settings, where breastfeeding duration is positively associated with income (Brion et al., 2011). Because mental health outcomes are negatively associated with socioeconomic status (Muntaner et al., 2004) most of these studies are subject to residual confounding. (Kramer et al., 2011) However, when the 1982 Pelotas birth cohort started, there was little awareness of the benefits of breastfeeding, in Brazil, and breastfeeding was independent of socioeconomic status. (Brion et al., 2011).

This study was aimed at assessing the association between breastfeeding, and mental health outcomes in young adults, including depression (major depression and depression severity), generalized anxiety disorder (GAD), social anxiety disorder (SAD) and common mental disorders (CMD).

## 2 Methods

In 1982, maternity hospitals in Pelotas, a southern Brazilian city, were visited daily and all births were identified. The 5914 liveborns whose families lived in the urban area of the city were examined and their mothers interviewed. These individuals have been followed in several occasions. (Barros et al., 2008) From June 2012 to February 2013, at a mean age of 30.2 years, we tried to follow the whole cohort and participants were invited to visit the research clinic to be interviewed and examined. (Horta et al., 2015b).

In the 30 years visit, we used the self-reported questionnaire (SRQ-20); validated for Brazil (Mari and Williams, 1986), to assess the presence of CMD. Males with a score of six or more and females with eight or more were considered as positive for CMD (Mari and Williams, 1986). In addition, we performed psychological interviews using the Mini-international psychiatric interview (MINI) V5.0 validated for Brazil (Amorim, 2000) and assessed the presence of a major depressive episode during the last two weeks, a lifetime episode of mania/hypomania, generalized anxiety disorder (GAD) in the last 6 months, and social anxiety disorder (SAD) in the last month. In addition, we used the Beck Depression Inventory (BDI-II) to evaluate the intensity of depressive symptoms.

We considered an individual as having major depression (MD) if a major depressive episode was reported in the last two weeks, and there was no evidence of a lifetime episode of mania/hypomania according to the MINI.

Those individuals whose BDI-II score was between 0 and 13 points were considered as minimal/no depression, mild depression was defined by a score of 14-19, moderate from 20 to 28 and severe from 29 to 63 points (Beck et al., 1996).

Information on duration of breastfeeding was gathered in the 1984 (mean age of 19 months) and 1986 (mean age of 42 months) visits, and the earliest available data on age at weaning was used to minimize recall bias. We divided the time of breastfeeding in four categories, less than 1 month, 1–2.9 months, 3–5.9 months, and 6 months or more.

Poisson regression with robust adjustment of the variance was used in crude and multivariable analysis to estimate prevalence ratios (PR) (Barros and Hirakata, 2003) of mental health outcomes using the MINI and CMD. For the BDI-II, we estimated the proportional odds ratios (OR) using ordinal regression and the Brant test was used to evaluate the proportional odds assumption.

Multivariable models were adjusted for the following confounding variables: sex; genomic ancestry (percentage of African ancestry); birth weight; type of delivery (information was gathered from the hospital records); maternal age at birth; maternal marital status (married/living with partner or not married); parental schooling (highest grade achieved at school); number of previous gestations; maternal smoking during pregnancy; family income at delivery; assets index; mother reference of nerve problems (proxy of mothers general mental health); father living in the same house and paternal history of psychiatric problems. Genomic ancestry was estimated from DNA samples that were genotyped using the Illumina Omni 2.5 M array (Illumina, San Diego, CA, USA). Admixture analyses were based on 37,0539 single nucleotide polymorphisms shared by samples from the HapMap Project, the Human Genome Diversity Project (HGDP), and the Pelotas cohort. The following HapMap samples were used as external panels: 266 Africans, 262 Europeans (American and Italian), 77 admixed Mexican Americans, 83 African Americans, and 93 Native Americans from the HGDP. For each individual, the proportion of European, African American, and Native American ancestry was estimated. (Lima-Costa et al., 2015) We tested the interaction between sex and breastfeeding over the evaluated outcomes, in multivariable models.

We obtained ethical approval for the study from the ethics committee in the 'Universidade Federal de Pelotas’, all participants signed an informed consent.

## 3 Results

In the 2012–13 visit, 3701 subjects were interviewed, which added to the 325 subjects known to have died, represented a follow-up rate of 68.1%. Of the 5914 livebirths included in the cohort, 5332 had information on breastfeeding, of these, 3524, 3459, 3456, and 3445 individuals, had information on CMD, MD, GAD, and SAD at 30 years, respectively. Data on at least one mental health outcome was available for 3661 individuals, 97% of which had information on duration of breastfeeding (n=3542). The follow-up rate at 30 years was higher among females, those whose mothers had between 5 and 8 years of schooling and whose family income ranged from 1 to 6 minimum wages. (Supplementary Table 1)Table 1 shows that 52% of the individuals included in the analysis were females; about one in every three subjects had a mother with four or less years of schooling at delivery; and one of every four were positive for common mental disorders and 12.7% had GAD. Prevalence of mild, moderate and severe depression was 10.9%, 7.8%, 5.2%, respectively. Prevalence of major depression according to the MINI was 7.9%.

Table 2 shows that the proportion of subjects who were breastfed for 6 months or more was independent of gender, family income and maternal schooling. On the other hand, females had a higher prevalence of mental disorders, and mental health was associated with maternal income and schooling.

Table 3 shows that compared to those breastfed for less than 1 month, individuals who were breastfed for 6 months or more had a lower prevalence of CMD (PR=0.84 [95%CI:0.71−0.98]). After controlling for confounders, the confidence intervals included the reference. Models evaluating major depression, showed similar results. In crude analyses, those subjects who breastfed for 6 or more months had a lower risk of major depression (PR=0.69 95%CI[0.51−0.95]). However, after controlling for confounders, the magnitude of the associations slightly decreased and the confidence intervals included the reference. The odds of having a more severe depressive symptoms (BDI-II) was smaller among those breastfed for 6 or more months even after controlling for confounding variables (OR=0.69 95%CI[0.53−0.89]). Concerning GAD and SAD, those who were breastfed for a longer time also showed a smaller risk of disease, however, we did not evidence any clear pattern of association and all confidence intervals included the reference (Table 3).

We did not evidence differences between the measures of associations in males and females, all interactions terms had a p-value >0.1, and for this reason, we did not stratified the analyses by sex.

## 4 Discussion

In this cohort followed since birth, in a southern Brazilian city, we found that individuals breastfed for more than 6 months were less likely to have more severe symptoms of depression, measured by the BDI-II. In addition, for most of the mental health outcomes, a longer duration of breastfeeding was associated with a lower risk of disease; however, the confidence intervals barely included the reference in most of the analysis. Therefore, we cannot exclude that these associations were due to chance. Because the prevalence of most of the outcomes was small, ranging from 3.6 to 12.7% (MINI diagnoses), the study might have been underpowered.

For this reason, we believe that our findings suggest that breastfeeding is associated with mental health in early adulthood, specially depression or depressive symptomatology. The pattern of association was similar for all depressive like outcomes, which was not the case for anxiety outcomes.

Studies evaluating the effect of breastfeeding over specific mental disorders are scarce. Most studies show the protective effect of breastfeeding over outcomes like general behavior (Heikkila et al., 2011, Liu et al., 2006, Liu et al., 2014, Yi et al., 2005) or mental well-being, (Reynolds et al., 2014) in children and adolescents. Among those few studies evaluating specific mental illness, the evidence is not conclusive. Allen et al. (1998) found that among those subjects who were never breastfed the odds of having major depression were higher, even in fully adjusted models (OR=1.64 [1.07–2.51]), but found no association with anxiety. However, Hayatbakhsh et al. (2012) found that breastfeeding for 4 or more months reduced anxiety/depression symptoms, of adolescents at age 14, but this association was reduced and confidence intervals included the reference in fully adjusted models. Similarly, Kwok et al. (2013) found that breastfeeding had no effect over depressive symptoms in a birth cohort of 11 years-old children.

In adults, Peus et al. (2012b) reported that the odds of being depressed were higher among those breastfed for less than 2 weeks. Similarly, Merjonen et al. (2010) found that breastfeeding decreased the inherent risk of depression present in individuals with the C/C genotype of the estrogen receptor 1 gene. On the other hand, Anselmi et al. (2008), in a previous study using the 1982 Pelotas birth cohort, observed that breastfeeding was not associated with common mental disorders at 23 years.

A possible pathway explaining the association between breastfeeding and mental health could be the relationship between breastfeeding and cognitive development. It has been shown in longitudinal studies that children with lower IQ have an increased risk of developing adult depression (Koenen et al., 2009), and a recent meta-analysis have shown that breastfeeding is associated with a higher IQ (Horta et al., 2015a). However, no previous study have formally tested the mediating effect of IQ. Nonetheless, eicosapentaenoic acid (EPA), present in human milk and one of the possible components involved in the mechanisms explaining the association between breastfeeding and IQ, along with docosahexaenoic acid (DHA), has been proposed as a diet supplement in the treatment of depression (Martins, 2009).

Some studies suggest that home environment and/or maternal care during childhood could influence the appearance of later mental health disorders (Gauthier et al., 1996, Oakley Browne et al., 1995, Rey, 1995), and that breastfeeding is associated with parent-child interaction qualities (Papp, 2014). However, in this study, we did not have information on home environment characteristics during childhood nor maternal-infant bonding, therefore we are unable to assess whether the protective effect of breastfeeding on mental health is attributable to the biological components of breast milk, to mother-infant bonding or a combination of both.

Concerning selection bias, the absence of differential follow-up with respect to breastfeeding duration, and the similar follow-up rates for several baseline characteristics suggest that the present results are unlikely to have been affected by selection bias. In addition, the negative results cannot be explained by a higher mortality in individuals with depression due to suicide. Among the 325 deaths that occurred, mortality due to suicide should be extremely low and did not underestimate the association. We showed in a previous report that 244 individuals died during the first 4 years of life, and 44 between 5 and 24 years, and only 19 of them died by external causes, mainly violence or accidents (Horta et al., 2008).

We should also mention that we did not ask for current psychological treatment in this follow-up, therefore some individuals with negative symptoms could actually be positive cases in treatment. However, in this follow-up, less than 3% (n=79), of those with no mental disorder according to the MINI, visited a psychiatrist during the last year. Therefore, we consider that this non-differential misclassification had a small effect in our estimates.

Information on duration of breastfeeding was collected at 19 months for 96% of the sample, and 42 months for the remainder, the weighted kappa comparing the information provided in 1984 and 1986 was 0.80, suggesting a high degree of agreement. A validation study done in a subsample of the cohort showed that 24% of mothers misclassified breastfeeding duration measured in 3-month categories, but in nearly all such cases misclassification involved neighboring categories (Huttly et al., 1990). Furthermore, it is important to bear in mind that any non-differential misclassification tends to decrease the magnitude of the associations. Therefore, the observed associations are unlikely to be due to recall bias.

Concerning residual confounding, we believe that the observed associations are not due to confounding by socioeconomic status, because in our cohort there was no clear social patterning of breastfeeding according to socioeconomic status. Furthermore, in the present study, adjustment for several possible socioeconomic and demographic confounders resulted in slight changes in the measures of association, whereas in the presence of residual confounding, adjusted estimated tend to be quite different in relation to the crude ones.

In summary, our findings suggest that breastfeeding reduces the odds of having more severe depressive symptoms. In addition, the effect of breastfeeding over other mental health outcomes might be small, reason why we were not able to find an association with the MINI diagnoses.

## Figures and Tables

**Table 1:** Sociodemographic characteristics at birth, breastfeeding and mental health outcomes at 30 years in individuals from the 1982 Pelotas birth cohort.

**Variable**	**N**^a^	**Mean (s.d.)/Median [IQR]**	**Prevalence %**
Female	1841		52
Mother’s with four or less years of schooling	1135		32.1
Mother’s age at birth (years)	3541	26.0 (6.2)	
Birth weight (grams)	3541	3224 (526)	

Breastfeeding			
<1 month	753		21.3
1–2.9 months	909		25.7
3–5.9 months	816		23.0
≥6 months	1064		30.0
CMD	853		24.1
MD	272		7.9
GAD	438		12.7
SAD	127		3.7

BDI-II			
Mild	375		10.8
Moderate	270		7.8
Severe	179		5.2

s.d.=standard deviation. IQR=Interquartile range. CMD=Common mental disorders. MD=Major depression. GAD=Generalized anxiety disorder. SAD=Social anxiety disorder. BDI=Beck depression inventory.

a Number of individuals with data on at least one mental health outcome and breastfeeding.

**Table 2:** Breastfeeding and mental health variables according to sociodemographic characteristics at birth.

	Breastfeeding at 6 months	CMD	MD	GAD	SAD
	N (%)	N (%)	N (%)	N (%)	N (%)
*Sex*	p=0.94	p<0.001	p<0.001	p<0.001	p<0.001
Male	510 (30.0)	360 (21.2)	70 (4.2)	118 (7.1)	34 (2.1)
Female	554 (30.1)	493 (27.0)	202 (11.3)	320 (17.8)	93 (5.2)
*Income tertiles of minimum wage*	p=0.63^a^	p<0.001^a^	p<0.001^a^	p<0.001^a^	p<0.001^a^
1	359 (32.4)	337 (30.4)	108 (10.0)	178 (16.4)	55 (5.1)
2	355 (28.2)	300 (24.0)	96 (7.9)	137 (11.2)	46 (3.8)
3	350 (29.9)	216 (18.5)	68 (5.9)	123 (10.7)	26 (2.3)
*Maternal schooling in years*	p=0.17^a^	p<0.001^a^	p<0.001^a^	p<0.001^a^	p<0.001^a^
0–4	366 (32.2)	324 (28.7)	104 (9.4)	178 (16.2)	57 (5.2)
5–8	418 (27.3)	387 (25.4)	119 (7.9)	179 (12.0)	47 (3.1)
9–11	99 (25.9)	76 (20.0)	29 (7.7)	45 (11.9)	12 (3.2)
≥12	179 (36.7)	63 (13.0)	19 (4.0)	35 (7.4)	10 (2.1)

a p-values using chi-squared test for trend. All other p-values were calculated using chi-squared tests for heterogeneity. CMD=Common mental disorders. MD=Major depression. GAD=Generalized anxiety disorder. SAD=Social anxiety disorder.

**Table 3:** Crude and multivariate models evaluating the association between breastfeeding and mental health outcomes at 30 years in the Pelotas birth cohort of 1982.

**Mental Disorder**				**Breastfeeding**			
			*p-Value*	<1 m	1–2.9 m	3–5.9 m	≥6 m
***CMD***	***Crude***	***PR (95%CI)***	p=0.18^b^	1	0.92 (0.79−1.09)	0.83 (0.70−0.99)	0.84 (0.71−0.98)
***Adjusted***^a^	***PR (95%CI)***	p=0.14^b^	1	0.94 (0.78−1.13)	0.91 (0.75−1.11)	0.87 (0.72−1.05)

***MD***	***Crude***	***PR (95%CI)***	p=0.08^c^	1	0.71 (0.51−0.97)	0.78 (0.57−1.08)	0.69 (0.51−0.95)
***Adjusted***^a^	***PR (95%CI)***	p=0.44^c^	1	0.79 (0.53−1.16)	0.93 (0.63−1.38)	0.77 (0.53−1.12)

***GAD***	***Crude***	***PR (95%CI)***	p=0.40^c^	1	0.88 (0.69−1.13)	0.91 (0.71−1.18)	0.81 (0.63−1.03)
***Adjusted***^a^	***PR (95%CI)***	p=0.23^c^	1	0.86 (0.64−1.16)	1.02 (0.77−1.37)	0.79 (0.59−1.06)

***SAD***	***Crude***	***PR (95%CI)***	p=0.54^c^	1	0.72 (0.44−1.17)	0.79 (0.49−1.29)	0.75 (0.48−1.20)
***Adjusted***^a^	***PR (95%CI)***	p=0.27^c^	1	0.61 (0.35−1.08)	0.90 (0.52−1.55)	0.66 (0.38−1.15)

***BDI-II***	***Crude***	***OR (95%CI)***	p=0.02^b^	1	0.89 (0.71−1.11)	0.85 (0.68−1.07)	0.77 (0.62−0.96)
***Adjusted***^a^	***OR (95%CI)***	p=0.01^c^	1	0.72 (0.55−0.94)	0.91 (0.70−1.20)	0.69 (0.53−0.89)

a Adjusted for sex, ancestry, birth weight, maternal age, maternal education, marital status, previous gestations, income at birth, smoking during pregnancy, type of delivery, assets index, mother nerve problems, father living together and father history of psychiatric illness.

b Test for linear trend.

c Wald test p-value for heterogeneity. CMD=Common mental disorders. MD=Major depression. GAD=Generalized anxiety disorder. SAD=Social anxiety disorder. BDI-II=Beck depression inventory.
