# Supplementary material for: Breastfeeding and mental health in adulthood: A birth cohort study in Brazil
Source: J Affect Disord. 2016 Sep 15;202:115–9. doi: 10.1016/j.jad.2016.05.055 (PMC4957542; doi:10.1016/j.jad.2016.05.055)
Supplement: Supplementary file 1 — Supplementary material [file mmc1.docx]

**Supplementary Table 1. Proportion of individuals from the original 1982 cohort with mental health data in 2012-13, according to selected characteristics**

|  | **Original cohort (number)** | **Followed at 30 years*** | **Followed at 30 years with mental health data**** |
| --- | --- | --- | --- |
| Gender |  | p<0.001 | p=0.82 |
| Male | 3037 | 66% | 96% |
| Female | 2876 | 71% | 97% |
| Breastfeeding |  | p=0.11 | p=0.95 |
| <1m | 1171 | 70% | 92% |
| 1-2.9m | 1405 | 69% | 93% |
| 3-5.9m | 1212 | 72% | 93% |
| ≥6m | 1544 | 72% | 96% |
| Family Income at birth (Minimum wages) | | p<0.001 | p=0.40 |
| ≤1 | 1288 | 67% | 95% |
| >1-3 | 2789 | 71% | 97% |
| >3-6 | 1091 | 70% | 96% |
| >6-10 | 382 | 61% | 98% |
| ≥10 | 335 | 60% | 98% |
| Maternal schooling |  | p<0.001 | p=0.99 |
| 0-4 | 1960 | 69% | 88% |
| 5-8 | 2454 | 71% | 91% |
| 9-11 | 654 | 66% | 93% |
| ≥12 | 839 | 63% | 97% |
| *Chi-squared p-value for heterogeneity between those followed at 30 years and the original cohort. Included 325 members know to have died. **Chi-squared p-value for heterogeneity between those followed and interviewed at 30 years and those with mental health data | | | |
